# New cytotoxic compounds from the leaves of *Caesalpinia* *benthamiana* (Baill.) Herend. & Zarucchi (Fabaceae)

**Published:** 2021

**Authors:** Tayo I. Famojuro, Taiwo O. Elufioye, Olumayokun A. Olajide, Michael Dybek, Adeboye Adejare

**Affiliations:** 1 *Department of Pharmacognosy, Faculty of Pharmacy, University of Ibadan, Ibadan, Nigeria*; 2 *Department of Pharmacy, School of Applied Sciences, University of Huddersfield, West Yorkshire, England*; 3 *Philadelphia College of Pharmacy, University of Sciences in Philadelphia, Philadelphia, PA 19104, USA*

**Keywords:** Cytotoxicity, Caesalpinia benthamiana, Benthamiacone, PC3, MCF-7, A549

## Abstract

**Objective:**

The incidence of multi-drug resistant cancer and the adverse effects associated with available chemotherapy have necessitated the search for new drug candidates. This study investigates the cytotoxic activity of *Caesalpinia benthamiana*.

**Materials and Methods:**

Column chromatography (CC) and preparative thin layer chromatography (PTLC) were used to isolate compounds. Structural elucidation was done by spectroscopic analysis. MTT assay was used to evaluate cytotoxicity of the compounds against three human adenocarcinoma cells, using methotrexate and dimethyl sulfoxide (DMSO) as positive and negative controls, respectively. CyQuant direct cell proliferation and caspase-3/7 green detection assays were used to investigate the dichloromethane fraction. IC_50_ values of isolated compounds were determined from sigmoidal dose-response curve.

**Results:**

Four new cytotoxic compounds, benthamianoate (2), benthamiacone (3), benthamianin (5) and benthamianol (6), and two known compounds, methyl gallate (1) and 2-methoxyacrylic acid (4) were identified. All the compounds were active with the new monoterpenoid characterized as benthamiacone exhibiting the highest activity (IC_50 _13.23-21.97 μg/ml) across cancer cell lines investigated. CyQuant direct cell proliferation assay showed significant reduction in the number of live carcinoma cells, while caspase-3/7 green detection assay showed significant increase in the number of dead carcinoma cells.

**Conclusion:**

This study revealed potential cytotoxic compounds which are here reported for the first time from *C. benthamiana*.

## Introduction

Cancer, a disease characterized by uncontrolled cell division resulting from prolonged unrepaired DNA damage by electrophilic species, can arise in any part of the body (WHO, 2018). Cancer is a main cause of death, with approximately 9.6 million cancer-related deaths and 18.1 million new cancer cases in 2018 (Bray et al., 2018). It is responsible for 1 out of 6 deaths recorded for all diseases annually (Hannah and Max, 2018) with the developing nations of the world accounting for approximately 56% of new cases and 70% of cancer mortality worldwide (Bray et al., 2018). 

In 2018, cancers of the lung, breast, colorectal, prostate, skin and stomach were the most commonly diagnosed ones worldwide, while the commonest mortality was due to cancers of tracheal, lung, colorectal, colon, stomach, liver and breast (WHO, 2018). Environmental factors such as physical, chemical, biological, and dietary carcinogens have been indicated as major causes of cancer disease (WHO, 2018). 

The genus *Caesalpinia*, is probably the smallest genera of the family Fabaceae, with about 200 species. This genus has been used in folkloric healthcare against numerous diseases and various investigators have validated their folklore uses (Zamble et al., 2008). *C. benthamiana* is widely distributed in West and Central Africa and is usually found in humid, rain forest, waste ground localities, deciduous woodland and savanna (Osho, 2014). In Africa traditional medicine, it has been used in treating erectile dysfunction, toothache and venereal diseases (Zamble et al., 2008), urethral discharge, snakebites, cataract and other eye problems (Dickson et al., 2006), dysentery (Irvine, 1961), skin diseases and wounds (Dickson et al., 2012). In addition, previous studies documented its anti- inflammatory (Moronkola et al*.*, 2009), antidiarrheal (Mbagwu and Adeyemi, 2008), antioxidant, antimicrobial (Dickson et al., 2006), antifertility and vasorelaxing activities (Zamble et al., 2008). 

**Figure 1:**
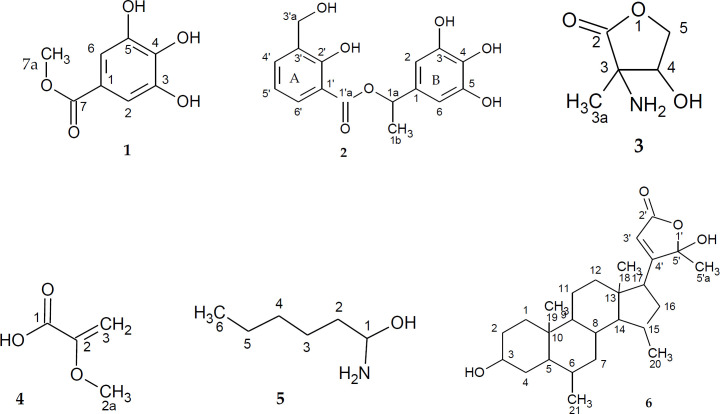
Compounds isolated from* C.* *benthamiana*

Furthermore, the antimicrobial activity of deoxycaesaldekarin C, benthaminin 1 and benthaminin 2 from the root, was reported (Dickson et al., 2006). The leaves were shown to contain monoterpenes, 36%; sesquiterpenoids, 19.6%; sesquiterpenes, 20.4% and a non-ubiquitous apocarotenoid-C_20_H_30_O, 16.7% (Olufunke et al., 2009). Other compounds such as methyl gallate, gallic acid, shikimic acid-3-*o*-gallate, epicatechin, 1-*o*-methyl-D-chiro-insotol, epicatechin-3-*o*-gallate, Kaepferol-3-(6-galloyl), benthaminin A-B, deoxycaesaldekarin C, β-sitosterol and stigmastenone were isolated from the root (Dickson et al., 2012). The current work reports the identification and cytotoxic activity of four new and two known compounds (Figure 1), isolated from the leaves of *C. benthamiana*.

## Materials and Methods

**Chemicals**

Solvents were purchased from Sigma-Aldrich, Germany. Cell culture materials were from Invitrogen, (Carlsbad, CA, USA). Methotrexate, penicillin-streptomycin suspension, trypan blue and the MTT salt were from Thermo Fisher Scientific (England). 

**General procedures**

Silica gel (80-200 mesh, Merck; Germany) was used for column chromatography (CC). Silica gel 60 F_254_ plates (10 x 5 cm, 0.5 mm; Merck, Germany) were used for thin layer chromatography (TLC). Silica gel 60 F_254_ plates (20 x 20 cm, 0.5 mm; Merck, Germany) were used for PTLC. PerkinElmer Fourier transform infrared spectroscopy (FTIR). Spectrum BX spectrometer was used for infra-red (IR) experiments. Mass data were obtained from the atmospheric pressure chemical ionization mass spectroscopy (APCI-MS). NMR spectra were recorded in CD_3_OD on Bruker Ascend^TM^ Spectrometer at 500 MHz (^1^H NMR) and 150 MHz (^13^C NMR). Distortionless Enhancement by Polarization Transfer (DEPT) experiments were used to differentiate different carbon atoms. ^1^H homonuclear connectivity was identified by Correlation spectroscopy (*COSY)*. Heteronuclear single quantum correlation (*HSQC)* helped to determine ^1^H-^13^C bound connectivity and HMBC used to determine two and three ^1^H-^13^C and ^13^C-^13^C bound connectivity. The MTT assay absorbance was recorded on Tecan F50 microplate reader **(**Thermo Fisher Scientific, England). 

**Plant material**

*Caesalpinia benthamiana* fresh leaves were collected at Ode Ekiti, Nigeria, in July 2015. Identification and authentication were done at Forest Herbarium Ibadan (FHI) where a voucher specimen (FHI 110847) was deposited. The leaves were air dried for two weeks under shade at room temperature (20-25ºC). The dried leaves were powdered and stored in air-tight container prior to use.

**Isolation procedure**

Powdered leaves of *C. benthamiana* (0.8 kg) was extracted using methanol at 25ºC for 72 hr and concentrated *in vacuo* to give crude extract (192 g). Crude extract was suspended in methanol-water (75:25) and partitioned successively using n-hexane and dichloromethane (DCM). Each partitioned fraction was concentrated to dryness to obtain 47.21 g of hexane, 38.63 g of dichloromethane and 101.00 g of aqueous fractions. About 20 g of DCM fraction was introduced into CC (80.0 x 3.5 cm) packed with 120 g of silica gel (80-200 mesh size) and successively eluted with solvent mixtures of increasing polarity. A total of 349 fractions obtained were pooled to 21 based on TLC profile. Pooled fraction 7 (2.18 g), (65% - 35% to 60% - 40% hexane: ethyl acetate) was purified by PTLC, mobile phase (petroleum ether: ethyl acetate: acetone 8:2:1) to give compound (1), a UV active single band, eluted with ethyl acetate. Pooled fraction 8 (0.92 g), (60% - 40% to 55% - 45% hexane: ethyl acetate), on further purification using PTLC, mobile phase (petroleum ether: ethyl acetate: acetone 8:2:1) indicated different bands, which on elution produced UV active; off white oily substance (4), black viscous solid substance (5), greenish brown oily paste (6). Pooled fraction 10 (1.66 g), (45% - 55% to 40% - 60% hexane: ethyl acetate), was subjected to short CC (40.0 x 1.5 cm) to obtain 45 pooled to 6 fractions. Pooled fraction 4 (0.14 g), (50% - 50% to 40% - 60% hexane: ethyl acetate), was further purified by PTLC, mobile phase (petroleum ether: ethyl acetate: acetone 8:2:1), two UV active bands gave; a yellowish brown viscous substance (2), and a brownish black viscous substance (3).

**Cell culture**

Cell lines of prostate (PC3), breast (MCF-7) and lung (A549) were purchased from European Collection of Authenticated Cell Cultures (ECACC), Public Health, England. They were cultured in T75 flasks containing Dulbecco's modified eagle m*edium (DMEM*) supplemented with 10% Fetal bovine serum (*FBS*) and 1% penicillin-streptomycin suspension. They were maintained in a humidified atmosphere, containing 5% CO_2_ at 37°C and changes in their morphology were checked using an inverted microscope (Evos Life Technology, UK) at magnifications X40 and X100 every 48 hr. Cells were sub-cultured and split in a 1:3 ratio into another flask when they reached confluence by washing with 0.02% Ethylene diamine tetra acetic acid (EDTA) in Phosphate-*buffered* saline (*PBS*)PBS and they were dissociated using 0.2% trypsin. A manual haemocytometer (Hausser Scientific Company, New York USA) was used to count the cells. 

**Fractions and compounds preparation**

Fractions and pure compounds were dissolved in DMSO to give 200 μg/ml stock solution and serial dilutions were prepared to produce 100 and 50 µg/ml (fractions) and 100, 50, 25 and 12.5 µg/ml (compounds). Methotrexate was used at 100, 50, 25 and 12.5 µg/ml. Final concentration of DMSO in each sample was less than 0.1% (Basar et al, 2015).

***In vitro*** **MTT assay**

Cytotoxicity was evaluated using MTT assay (Deepa et al., 2011). Cells were seeded into 24-well plates and incubated at 37°C in humidified CO_2_ (5%) for 24 hr after which the old medium was replaced with serum-free FBS. Then, 0.2 μl of test compounds in triplicates was added onto the well containing cells except the blank well. The plates were again incubated for 24 hr with DMSO and methotrexate used as negative and positive control, respectively. Thereafter, old medium was replaced with 200 μl MTT solution (final concentration of 0.5 mg/ml in PBS). Plates were incubated again for 4 hr after which the MTT solution was removed and formed formazan crystals were dissolved by adding 150 μl DMSO. Absorbance was measured at 570 nm and percentage of cell viability was calculated as [(absorbance of treated cells- absorbance of blank) / (absorbance of control cells- absorbance of blank)] ×100).

**CyQuant®** **direct cell proliferation assay**

Dichloromethane fraction at 50 and 100 µg/ml was evaluated against MCF-7, A549 and PC3 using CyQuant® direct assay (Abraham et al., 2008). About 2000 cells were seeded in 48-well plates containing 400 μl of medium and incubated for 48 hr after which 0.2 μl of the fraction was added. Plates were incubated for another 24 hr and 200 μl of supernatant aspirated from each well was replaced with 200 μl of 2X detection reagent and plates were incubated again at 37°C for 60 min. Cells images were obtained using standard green filter microscope. 

**Caspase-3/7 green detection assay**

Dichloromethane fraction (50 and 100 µg/ml) was evaluated using caspase-3/7 detection assay (Cobanoglu et al., 2016). About 2000 cells were seeded in 48-well plates containing 200 μl of medium and incubated for 48 hr after which 0.2 μl of the fraction was added. Plates were incubated at 37°C for 24 hr and the medium was replaced with 200 μl of caspase-3/7 green detection reagent. Plates were incubated again at 37°C for 30 min and imaging was done using standard microscope. 

**Statistical analysis**

All experiments were done in triplicate and results are expressed as mean±standard errors of means. Statistical analysis was performed using GraphPad (Version 7.0, GraphPad Prism Software Inc., San Diego, CA). Sigmoid dose-response curve obtained from non-linear regression analyses (log of inhibitor versus normalized response) was used to determine the IC_50_. One-way ANOVA with Dunnett’s multiple comparison tests was conducted at p*<*0.05 to detect difference in cytotoxicity of isolated compounds compared with methotrexate. Viability reducing ability of the isolated compounds was also compared with the negative control. Differences in the activity lower than p<0.05 were considered statistically significant.

## Results

**Characterization of isolated compounds**

Gradient CC and purification of the DCM fraction led to isolation of compounds 1-6 (Figure 1). 

Compound 1 (900 mg) was isolated as white/off-white crystalline powder, R_f _0.28 (petroleum ether/acetone/ethyl acetate 7:3:1), melting point 201°C (Lit m.pt 198–200°C), (Sanchez et al., 2013). APCI-MS m/z 184.9 [M^+^+H] corresponding to C_8_H_8_O_5_ (184.1491 calculated).^ 1^H NMR and ^13^C NMR data is reported in Table 1. Assignments were confirmed with 2D experiments and literature data (Sanchez et al., 2013). Compound 1 was identified as methyl gallate.

Compound 2 (130 mg) was isolated as a viscous yellowish brown substance, R_f _0.42 (petroleum ether/acetone/ethyl acetate 7:3:1), melting point 55°C. APCI-MS m/z 320.09 [M^+^+H] corresponding to C_16_H_16_O_7_ (320.28964 Calcd). ^1^H NMR and ^13^C NMR data is reported in Table 2. Assignments were confirmed with 2D experiments. Compound 2 was identified as 1-(3, 4, 5-trihydroxyphenyl) ethyl 2-hydroxy-3-(hydroxylmethyl) benzoate (benthamianoate).

Compound 3 (200 mg) was isolated as a brownish-black viscous substance, R_f _0.69 (petroleum ether/acetone/ethyl acetate 7:3:1), melting point of 45°C. APCI-MS m/z 132.98 [M^+^+H] corresponding to C_5_H_9_O_5_ (13166 Calculated).^ 1^H NMR and ^13^C NMR data is reported in Table 3. 

Assignments were confirmed by 2D experiments. Compound 3 was identified as 3-amino-4-hydroxy-3-methyldihydrofuran-2-one (benthamiacone).

**Table 1:** ^1^H-and ^13^C NMR data for compound C1

**CB1 data**					**(Sanchez ** ***et al*** **., 2013)**	
**S/N.**	**Types of C**	_H_	_C _	**HMBC**	_H _	_C _
1	CQ	-	136.87	-	-	119.32
2	CH=	6.94 (1H, s, H-2)	109.06	136.87, 146.03, 109.06	6.94, s	107.86
3	CQ	-	146.03	-	-	144.10
4	CQ	-	136.87	-	-	136.99
5	CQ	-	146.03	-	-	144.10
6	CH=	6.94 (1H, s, H-6)	109.06	136.87, 146.03, 109.06	6.94, s	107.86
7	C=O	-	166.78	-	9.21, s	165.67
7a	CH_3_-O	3.75 (s)	52.15	166.78	3.74, s	50.36

**Table 2:** ^1^H-and ^13^C- NMR data for compound C2

**S/N**	**Types of C**	_H_	_C_	**HMBC**
1	CQ	-	131.60	-
2	CH=	6.91 (s)	109.20	73.10, 131.60, 138.40, 146.10
3	CQ	-	146.10	-
4	CQ	-	138.40	-
5	CQ	-	146.10	-
6	CH=	6.91 (s)	109.20	73.10, 131.60, 138.40, 146.10
1°	CH-	4.36-4.38 (m)	73.10	167.96
1b	CH_3_-	1.27 (s)	21.74	73.10, 167.96
1'	CQ	-	119.50	-
2'	CQ	-	151.0	-
3'	CQ	-	121.0	-
3'a	CH_2_-	3.75-3.81 (t)	71.97	-
4'	CH=	7.07 (d)	118.8	71.97, 114.0, 116.10
5'	CH=	7.03 (s)	116.10	118.8, 114.0
6'	CH=	7.09 (d)	114.0	116.10, 118.8
1ʹa	C=O	-	167.96	-

**Table 3:** ^1^H-and ^13^C- NMR data for compound C3

**S/N**	**Types of C**	_H_	_C_	**HMBC**
-	-	-	-	-
2	C=O	-	178.70	-
3	CQ-	-	72.03	-
3°	CH_3_-	1.25 (d)	21.74	72.03, 178.70, 73.17
4	CH-OH	4.01 (t)	73.17	72.03, 62.91
5	CH_2_-	4.3 (m)	73.10	167.96

Compound 4 (180 mg) was isolated as off-white oily crystals, R_f _0.43 (petroleum ether/acetone/ethyl acetate 7:3:1), melting point of 14°C. IR (KBr) υ_max_ 3461.5, 3286.5, 3087.1 (O-H), 2951.8 (CH_3_), 2850.0 (CH_2_), 1691.8 (C=O) cm-^1^. MS m/z 102.0 [M^+^+H] corresponding to C_4_H_6_O_3 _(102.09044 Calcd). ^1^H NMR and ^13^C NMR data is reported in Table 4. Assignments were confirmed by 2D experiments. Compound 4 was identified as 2-methoxyacrylic acid.

**Table 4:** ^1^H-and ^13^C- NMR data for compound C4

**S/N**	**Types of C**	_H_	_C_
1	C=O	-	168.27
2	CQ	-	145.18
2°	CH_3_-O	3.89 (s)	50.10
3	CH_2_=	5.52 - 5.50 (m)	108.86

Compound 5 (20 mg) was isolated as yellowish-brown crystals, R_f _0.20 (petroleum ether/acetone/ethyl acetate 7:3:1), melting point of 17°C. IR (KBr) υ_max_ 3362.3 (O-H), 2923.8 (CH_3_), 2853.6 (CH_2_), 1709.2 (C=O), 1512.4, 1443.9 (C-N), 1371.3, 1239.6 (C-O-C), 908.9 and 729.6 (C-H) cm-^1^. MS m/z 117.12 [M^+^+H] corresponding to C_6_H_15_NO (117.19 Calcd).^ 1^H NMR and ^13^C NMR data is reported in Table 5. Assignments were confirmed using 2D experiments. Compound 5 was identified as 1-aminohexane-1-ol (benthamianin).

Compound 6 (10 mg) was isolated as a greenisy-brown paste, R_f _0.36 (petroleum ether/acetone/ethyl acetate 7:3:1), melting point of 153°C. IR (KBr) υ_max_ 2916 (CH_3_), 2849 (CH_2_), 1734 (C=O), 1437 (CH-OH), 1373, 1251 (C-O-C) 913, 881, 757 (C-H) cm-^1^. MS m/z 416.29 [M^+^+H] corresponding to C_26_H_40_O_4_ (416.5878 Calcd).^ 1^H NMR and ^13^C NMR data is reported in Table 6. Assignments were confirmed by 2D experiments. Compound 6 was identified as 4-(hexadecahydro-3-hydroxy-6, 10, 13, 15-tetramethyl-1*H*-cyclopenta[*a*]phenanthren-17-yl)-5-hydroxy-5-methylfuran-2(5*H*)-one (benthamianol).

**Table 5:** ^1^H-and ^13^C- NMR data for compound C5

**S/N**	**Types of C**	_H_	_C_	**COSY**
1	CH-	4.17-4.39 (m)	76.71	H-2, H-3
2	CH_2_-	1.57-1.73 (m)	31.94	H1, H-3, H-4
3	CH_2_-	1.14-1.35 (m)	29.37	H-1. H-2, H-4, H-5
4	CH_2_-	1.14-1.35 (m)	29.71	H-2, H-3, H-5
5	CH_2_-	1.32-1.35 (m)	22.71	H-3, H-4, H-6
6	CH_3_-	0.97 (t)	14.22	H-4, H-5

**Bioactivity of fractions/compounds**

The cytotoxicity results of fractions and compounds from *C. benthamiana* evaluated using MTT assay against MCF-7, A549 and PC3, are presented in Tables 7 and 8. DCM fraction exhibited the highest activity (IC_50_=8.45±0.11 μg/ml) on PC3 with a moderate effect (IC_50_=10.46±0.10 μg/ml) on MCF-7 and a low effect on A549 with IC_50 _27.90±0.04 μg/ml. Hexane fraction had the least activity with IC_50_ values of 72.75±0.18, 12.61±0.16, and 31.07±0.07 μg/ml on MCF-7, A549 and PC3, respectively while aqueous fraction showed a moderate effect (Table 7). 

**Table 6:** ^1^H-and ^13^C- NMR data for compound C6.

**S/N.**	**Types of C**	_H_	_C_	**HMBC**
1	CH_2-_	1.23-1.57 (m)	31.9	23.7, 43.6, 75.1
2	CH_2-_	1.65-1.92 (m)	23.7	31.9, 30.0, 75.1
3	CH-OH	3.19 (bs)	75.1	30.0, 23.7, 31.9, 44.5
4	CH_2-_	1.57-1.92 (m)	30.0	44.5, 75.1, 43.6, 41.3, 23.7
5	CH-	1.65-1.92 (m)	44.5	75.1, 30.0, 41.3, 43.6, 27.7
6	CH-	1.31-1.58 (m)	41.3	30.0, 44.5, 43.6, 27.7, 37.3
7	CH_2-_	1.65-1.92 (m)	27.7	44.5, 41.3, 27.7, 37.3, 49.8
8	CH-	1.31-1.58 (m)	37.3	41.3, 43.6, 27.7, 37.3, 49.8, 50.9, 37.0
9	CH-	1.31-1.58 (m)	49.8	43.6, 27.7, 37.3, 31.9
10	CQ-	-	43.6	-
11	CH_2-_	1.27-1.61 (m)	22.2	43.6, 49.8, 37.3, 36.3
12	CH_2-_	1.23-1.57 (m)	36.3	22.2, 49.8, 36.7, 50.9, 49.7
13	CQ-	-	36.7	-
14	CH-	1.23-1.57 (m)	50.9	27.7, 37.3, 36.7, 50.9, 49.7, 31.6
15	CH-	1.65-1.92 (m)	37.0	37.3, 36.7, 50.9, 49.7, 31.6, 14.5
16	CH_2-_	1.31-1.58 (m)	31.6	36.7, 50.9, 49.7, 37.0, 14.5
17	CH-	2.12-2.17 (m)	49.7	115.6, 170.1, 108.0, 36.3, 36.7, 31.6
18	CH_3-_	1.23 (s)	17.6	36.7, 49.7
19	CH_3-_	1.25 (s)	11.9	43.6, 49.8, 31.9
20	CH_3-_	1.18 (s)	14.5	50.9, 37.0, 31.6
21	CH_3-_	1.16 (s)	14.2	44.5, 41.3, 27.7
1ʹ	-	-	-	-
2ʹ	C=O	-	171.4	-
3ʹ	CH=	5.8 (s)	115.6	170.1, 108.0, 171.4, 49.7
4ʹ	CQ=	-	170.1	-
5ʹ	CQ-	-	108.0	-
5ʹa	CH_3_	1.81 (s)	21.2	170.1, 108.0

**Table 7:** Cytotoxic effect of *C. benthamiana* fractions

**Fractions**	**IC**_50_ ** (µM)**		**95% CI**		
**MCF-7 cell**					
Hexane	72.75±0.18	22.18 to 244.9			
DCM	10.46±0.10	6.136 to 16.67			
Aqueous	11.28±0.08	7.322 to 16.47			
Methotrexate	12.11±0.06	8.726 to 16.24			
**A549 cell**					
Hexane	12.61±0.16	5.134 to 25.42			
DCM	8.45±0.11	5.07 to 13.52			
Aqueous	21.17±0.07	14.06 to 30.03			
Methotrexate	9.97±0.08	6.576 to 14.52			
**PC3 cell**					
Hexane	31.07±0.07	20.26 to 45.27			
DCM	27.90±0.04	22.16 to 34.53			
Aqueous	16.60±0.04	12.98 to 20.74			
Methotrexate	12.98±0.07	8.851 to 18.14			

^a^MCF7=breast adenocarcinoma; A549=lung adenocarcinoma; and PC3=prostate adenocarcinoma.

^b^MTT assay, (means±SEM, n=3), CI=confidence interval.

The DCM fraction was purified using CC and PTLC to afford compounds 1-6. Cytotoxicity assay showed that all compounds had significant effects, with benthamiacone (3) being the most active [(IC_50_ value=21.97±0.06 μg/ml; R^2^=0.83, 13.46±0.07 μg/ml; R^2^=0.87 and 13.23±0.10 μg/ml; R^2^=0.70)] against breast, lung and prostate carcinoma cells, respectively, while C1, C2, C4 and C6 showed moderate cytotoxicity. The lowest activity (IC_50=_63.87±0.17 μg/ml, R^2^=0.09) was observed for methoxyacrylic acid (C5) (Table 8).

**CyQUANT®** **direct cell proliferation and caspase-3/7 green detection assays**

**Figure 2:**
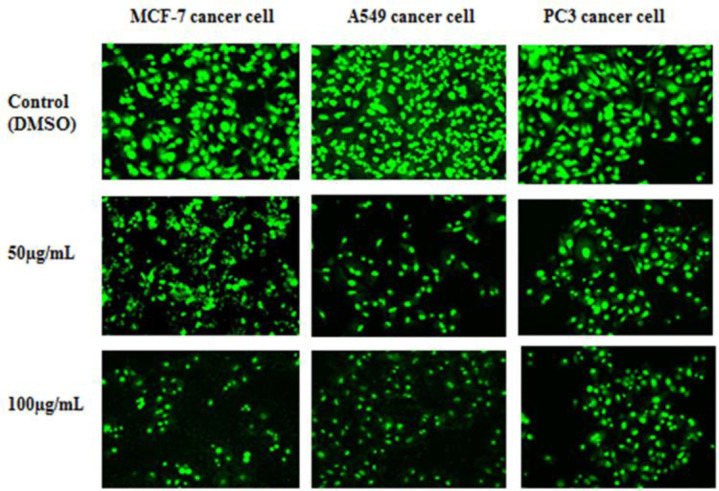
Photomicrographs showing the effect of the dichloromethane fraction (50 and 100 μg/ml) on the proliferation of MCF-7, A549 and PC3 adenocarcinoma cells using cyQuant direct cell proliferation assay, where green colours show reduction of viable cells proliferation. Cells images were acquired on a standard FITC filter set microscope (X100 magnification). Scale bar = 100 μm

**Figure 3:**
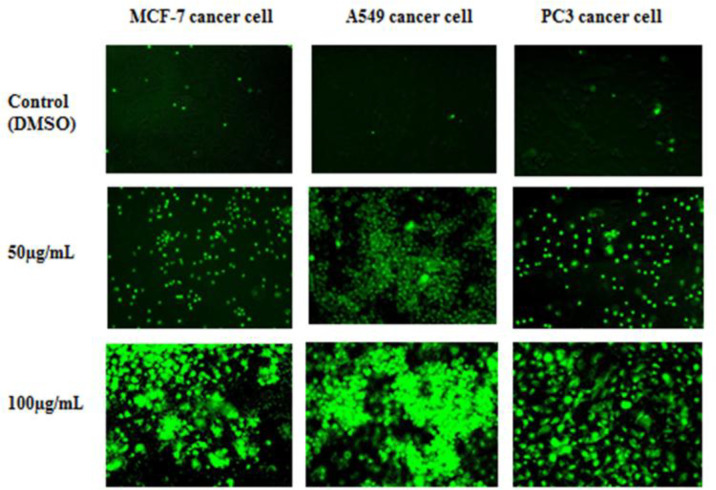
Photomicrographs showing the effect of the dichloromethane fraction (50 and 100 μg/ml) on MCF-7, A549 and PC3 adenocarcinoma cells using caspase-3/7 assay, where bright fluorogenic green colours indicate dead cells. Cells imaging was done on a standard FITC filter set microscope (X100 magnification). Scale bar = 100 μm

In addition to MTT assay, the DCM fraction was evaluated by CyQuant® direct cell proliferation and caspase 3/7 green detection assays. The results are presented in Figures 2 and 3 respectively.

**Table 8:** Cytotoxic effect of isolated compounds from* C. benthamiana*

**Compounds **	**IC**_50_ ** (µg/mL)**	**95% CI**	
**MCF-7 cell**			
C1	13.44±0.17	4.512 to 35.11	
C2	26.44±0.12	12.59 to 54.77	
C3	21.97±0.06	15.36 to 31.01	
C4	31.37±0.70	21.69 to 45.29	
C5	63.87±0.17	21 to 221	
C6	7.197±0.25	1.666 to 24.23	
Methotrexate	8.261±0.08	5.267 to 12.42	
**A549 cell**			
C1	28.97±0.09	16.45 to 50.63	
C2	16.16±0.10	9.144 to 26.98	
C3	13.46±0.07	9.115 to 19.35	
C4	22.09±0.14	8.203 to 55.37	
C5	16.33±0.10	9.519 to 26.92	
C6	10.9±0.14	4.483 to 23.33	
Methotrexate	9.734±0.15	5.106 to 17.14	
**PC3 cell**			
C1	8.44±0.05	6.429 to 10.9	
C2	11.13±0.18	3.432 to 29.69	
C3	13.23±0.10	7.426 to 23.3	
C4	16.71±0.13	8.994 to 23.32	
C5	10.78±0.10	8.645 to 13.3	
C6	10.29±0.04	8.237 to 12.65	
Methotrexate	7.054±0.08	4.661 to 10.3	

^a^MCF7=breast adenocarcinoma; A549=lung adenocarcinoma; and PC3=prostate adenocarcinoma.

^b^MTT assay, (means±SEM, n=3); CI=confidence interval.

## Discussion

The ^1^H/^13^C NMR spectra of C1, (methyl gallate) revealed an aromatic system exhibiting signals due to a pyrogallol ring at 6.94, 2H, (s) assigned to the chemically equivalent protons H-2/H-6 on carbons C-2/C-6 (δc 109.06). The methoxyl group at position C-7 was confirmed by HMBC correlation of a singlet OCH_3_ proton at 3.75 Hz (δc 52.15) to the carbonyl C-7 (δc 166.78). The ^13^C NMR spectra revealed three phenolic groups attached to C-3, C-4, and C-5. The signal of chemically equivalent C-3 and C-5 appeared more downfield at (δ 146.0) compared with phenolic carbon at C-4 (δ 138.67) due to the combination of inductive and anisotropic effects of the ring on the carbon atom. This spectroscopic data is in agreement with literature (Sanchez et al., 2013).

The ^1^H/^13^C-NMR data of C2, 1-(3, 4, 5-trihydroxyphenyl) ethyl 2-hydroxy-3-(hydroxymehtyl) benzoate (benthaminoate) revealed it as a benzoate derivative. Total assignment was possible from HMQC and HMBC data. The ^1^H NMR showed five aromatic protons with a chemical shift at 6.99 2H, (s) assigned to the chemically equivalent protons H-2/H-6 on carbons C-2/C-6 (δC 109.20) in ring (B); δ 7.07 (d, 5.75 Hz, H-4ʹ); 7.03 (s, H-5ʹ); and 7.09 (d, 5.75 Hz, H-6ʹ) on carbons C-4ʹ, C-5ʹ and C-6ʹ (δC 118.8, 116.10 and 114.0) respectively in ring A. There was also a non-aromatic oxygenated methylene at δ_H_ 3.81 (δc 71,97) attached to C-3ʹ) of ring A, a methyl group at δH 1.75 (3H, s) attached to a non-aromatic oxygenated methine at δ_H_ 3.60-4.01 (δc 21.74) attached to C-1a (δc 73.10); The ^13^C NMR spectrum showed carbonyl carbon at δC 167.96 attached to C-1ʹ (δc 119.50) of ring A. In the ^13^C NMR, the six signals δc 119.50, 151.0, 121.0, 118.80, 116.10 and 114.0 (Table 2) were attributable to the carbons 1ʹ, 2ʹ, 3ʹ, 4ʹ, 5ʹ and 6ʹ of ring A while a chemical shift at δc 131.6 was assigned to C-1, δc 109.20 assigned to the chemically equivalent carbons (C-2/C-6), δc 146.10 assigned to another chemically equivalent carbons (C-3/C-5) and δc 138.40 assigned C-4 of ring B. The HSQC spectrum gave clear correlations of the carbon atoms with the protons directly attached to them. In the HMBC spectrum, the position of the methyl group at C-1b, was confirmed by the correlation of the methyl protons at 1.27 ppm with carbon C-1a (δc 73.10) and C-1ʹa δc 167.96) attributed to the carbonyl group. The signal δ_H_ 7.07 (H-4ʹ) was similarly situated with observed correlations of the aromatic proton with oxygenated methylene signal at C-3ʹa (δc 71.97) and C-5ʹ (δc 116.10), C-6ʹ (δc 114.0). In addition, H-2 and H-6 (δc 109.20) showed HMBC correlations with carbons C-1, C-3, C-4, C-5 and C-1a (δc 131.6, 146.10, 138.40 and 73.10). These assignments were clearly confirmed by COSY and DEPT experiments.

**Figure 4:**
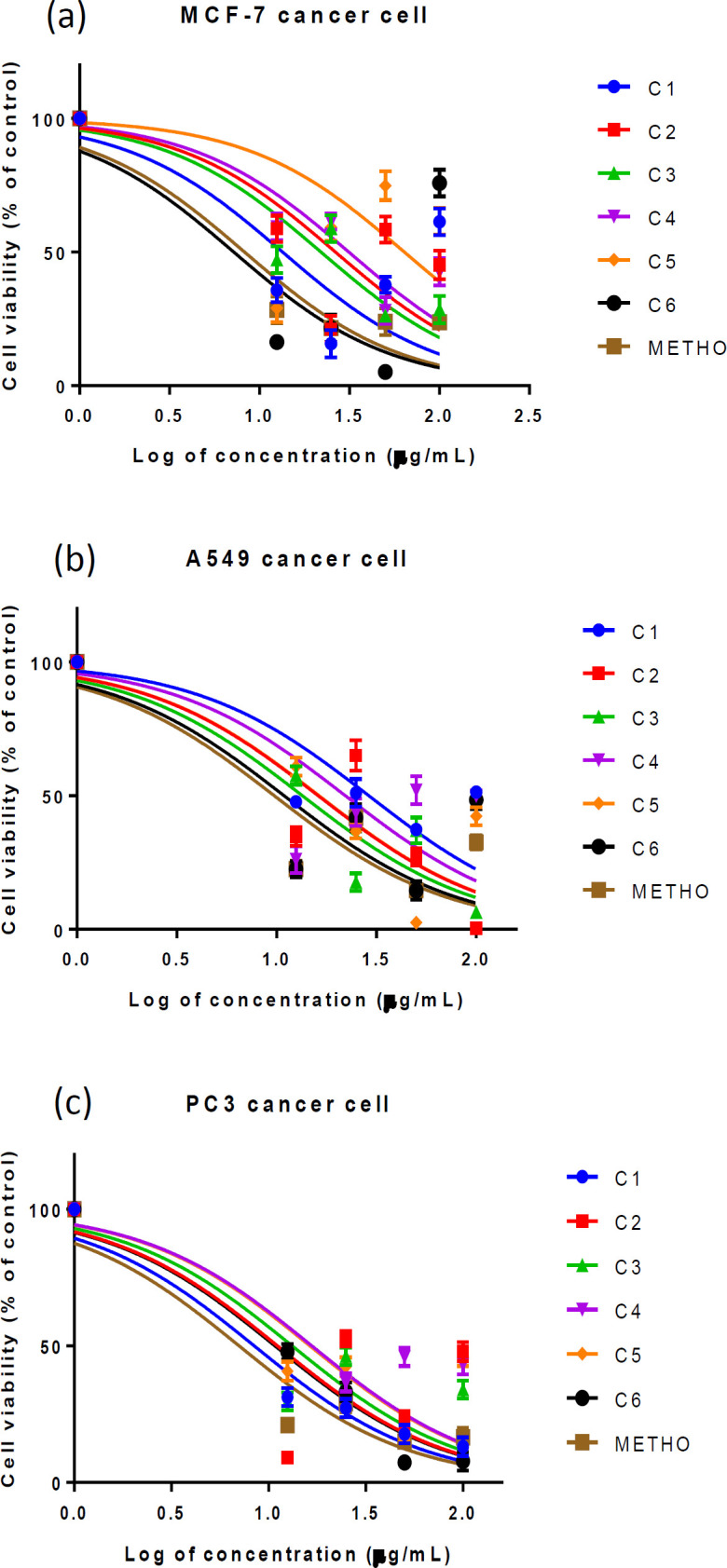
Dose-response curve for isolated compounds compared with methotrexate (standard reference) against (a) MCF-7, (b) A549 and (c) prostate adenocarcinoma cells. Absorbance values are in triplicate and the data was analyzed at p<0.05. Anti-log of concentration at 50% viability was taken as half-maximal inhibitory concentration (IC_50_) for each of the compounds

The^ 13^C NMR spectra of C3 (132.9 (M^+^+H)), 3-amino-4-hydroxy-3-methyldihydrofuran-2-one (benthamicone) indicated 5 carbon atoms. The most desheilded signal identified as carbonyl (δ 178.60) was assigned to C-2 and confirmed by DEPT spectra. ^13^C NMR further revealed an oxygenated methylene at δc 62.91 assigned to C-5, an oxygenated methine carbon at δc 73.17 assigned to C-4, a quaternary carbon at δc 72.03 (C-3) attached to a primary amine and a methyl group at δc 21.74 assigned to C-6. The ^1^H NMR spectra showed a methyl proton at δ_H_ 1.25 attached to C-3; an oxygenated methine proton at δ_H_ 4.01 attached to C-4; and an oxygenated methylene proton at δ_H_ 4.37 attached to C-5. In the HMBC, the position of the methyl group at 3a was confirmed by the correlation of the methyl protons at 1.25 ppm with carbon C-3 (δc 72.03); C-4 (δc 73.17); and C-5 (δc 62.91). These assignments were confirmed by COSY, HSQC, HMBC and DEPT.

The^ 13^C NMR spectra of C4 (2-methoxyacrylic acid) indicated quaternary carbons at δc 168.27 and δc 145.18 assignable to C-1 and C-2, respectively; methoxy carbon with a chemical shift δc 50.10; and one sp2 methylene carbon at δc 108.86. The ^1^H NMR spectrum revealed a singlet methoxy proton at δ_H_ 3.89 and a geminal proton signals at δ_H_ 5.52-5.50 (m) located on carbon C-3 (δc 108.86) as confirmed by DEPT and COSY. The carbonyl and hydroxyl groups were further confirmed by the IR spectra with signals at 1691 cm^-1^ and 3087 cm^-1^ , respectively.

The ^13^C NMR and DEPT spectra of C5, 1-aminohexane-1-ol (benthamianin) showed 6 carbons composed of a methyl, CH_3_ at C-6 (δc 14.22); four methylene CH_2_ at C-5 (δc 22.71), C-4 (δc 29.71), C-3 (δc 29.37) and C-2 (δc 31.94); oxygenated methine, HC-OH at position C-1 (δc 76.71) attached to a primary amine group. The observed ^1^H-^1^H COSY indicated the correlations of H-1 to CH_2_ of H-2, H-3; H-2 to H-3, H-4 and H-5; H-3 to H-4, H-5 and H-6; H-4 to H-5 and H-6; H-5 to H-6; H-6 cross peak H-5; H-5 cross peak H-4); H-4 cross peak H-3); H-3 cross peak H-2); and H-2 cross peak H-1. These assignments were confirmed by IR signals at 2923, 2853, 1709, 1443, 1371, 1239, 908 and 729 cm^-1^ which are characteristics of C-O, C-N, and C-H, respectively. ^1^H NMR showed a strong downfield signal at δ 4.19 (m), which could be attributed to the electronegative environment. Other signal from the ^1^H NMR spectra are δ 1.62 (m), δ 1.27 (m) δ 1.27 (m), δ 1.27 (m), and δ 0.89 (m).

The ^1^H/^13^C NMR of C6, 4-(hexadecahydro-3-hydroxy-6, 10, 13, 15-tetramethyl-1*H*-cyclopenta[*a*] phenanthren-17-yl)-5-hydroxy-5-methylfuran-2(5*H*)-one (benthamianol) showed one olefinic proton as a singlet at δ_H_ 5.80 (δc 115.8) assignable to C-3ʹ, an oxygenated methine, δH 3.19 (δc 75.1) assignable to C-7, doubly oxygenated carbon (hemiketal) assignable to C-5ʹ (δc 108.0), a quaternary carbonyl at δc 171.4 assignable to C-2ʹ. The ^13^C and DEPT spectra showed 26 carbon signals composed of CH_3_ (5), CH_2_ (7), non-olefinic CH (7), oxygenated methine, CHOH (1), olefinic CH (1) and quaternary carbons (5). The HMBC spectrum also showed the diagnostic connectivity of the olefinic proton at δ_H_ 5.80 (H-3ʹ) to C-2ʹ (δc 171.4), C-4ʹ (δc 170.1), C-5ʹ (δc 108.0), C-17 (δc 49.7). Crucial is also the HMBC connectivity of H-3 (δ_H_ 3.19) to C-2 (δc 23.7), C-1 (δc 31.9), C-4 (δc 30.0) and C-5 (δc 44.5). The identity of this compound was confirmed by DEPT, HSQC and HMBC. 

**Figure 5.**
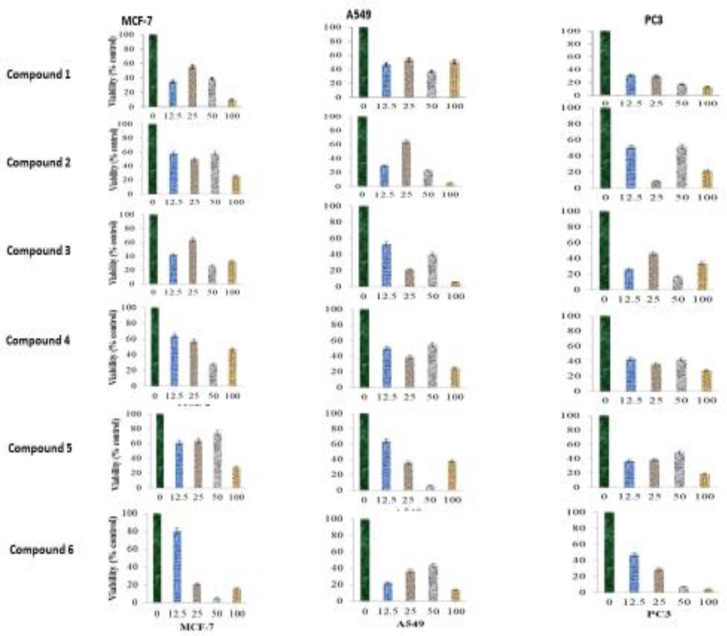
Induced reduction ability of (a) compound 1, (b) compound 2, (c) compound 3, (d) compound 4, (e) compound 5 and (f) compound 6 isolated from *Caesalpinia benthamiana* on the MCF-7, A549 and PC3 cells viability. Methotrexate (standard reference, IC_50 _ranged from 9.97 to 12.98 μg/ml). Values are means±SEM, n= 3), ***p<0.05 in comparison with the negative control (only DMSO) on x-axis, concentration in μg/ml

The choice of cancer cells for this study was done based on high occurrence and the need for effective medication. These cancer types are the commonest cause of death (Hannah and Max, 2018). Breast cancer is common in females while prostate cancer is the most commonly diagnosed in males (Popoola et al., 2013). In 2012, lung cancer accounted for 1.7 million deaths (18.2%) of the total cancer mortality (Torre et al., 2015) and 1.8 million deaths (18.4%) in 2018 (WHO, 2018).

In this study, three assays were used to assess fractions and compounds from *C. benthamiana. *The MTT assay showed DCM fraction as the most active on lung cancer cells followed by aqueous and hexane fractions (Table 7). Further investigation of DCM fraction by CyQUANT® direct assay showed reduction in the viability of cells and a progressive loss of cell membrane integrity (Figure 2). This dye is confirmed effective in evaluating anti-proliferative and cytotoxicity activity of drugs on cancer cells with the advantage of non-dependence on cell metabolic state (Quent et al., 2010). The caspase 3/7 assay also corroborated the observed activity of the DCM fraction. The fraction induced apoptosis in treated cells as shown by an increase in the number of dead cells. This was evident by a bright, fluorogenic green coloured dead cells in the treated groups compared to the untreated group (Payne et al., 2013). In some cases, a total rupture of the cell membrane was also observed (Figure 3).

Compounds isolated were phenolic acids (1, 2 and 4), alkaloid (5) and terpenoids (3 and 6). The terpenoids displayed the highest, phenolic acids moderate, while the alkaloid had the least activity. Our study showed that the isolated compounds had cytotoxic activity with benthamiacone (C3) (IC_50_ value=21.97±0.06, 13.46±0.07 and 13.23±0.10 μg/ml) against MCF-7, A549 and PC3, respectively having the highest antiproliferative effect. Generally, terpenoids and their derivatives are known with potent cytotoxic/antiproliferative activities (Rabi and Bishayee, 2009). 

The IC_50_ values of fractions and compounds indicated their inhibitory ability. These values were considered with the 95% confidence interval (CI) of the observed data (Tables 7 and 8). The CI is a function of the reliability of the IC_50 _generated from the data fitting to a dose-response curve (Labelle et al., 2019). The calculated inhibitory concentration obtained from the dose-response curve clearly demonstrated the cytotoxic potentials of the fractions and the isolated compounds investigated compared with the standard drug.

To the best of our knowledge, this is the first report of isolation and assessment of the cytotoxicity of methyl gallate (1), benthamianoate (2) and 2-methoxyacrylic acid (4), benthamianin (5) benthamiacone (3) and benthamianol (6) from *C. benthamiana*. Methyl gallate was previously reported as an anticancer, anti-inflammatory and antioxidant agent (Sanchez et al., 2013). Its cytotoxic activity was also supported by Kamathama et al., (2015) with methyl gallate inhibiting A431 cells. The compound also reduced tumor growth *in vitro* and in tumor-bearing mice by decreasing CD4^+^CD25^+^ Treg cell migration and reducing the suppressive function of effector T cells (Lee et al., 2010). Furthermore, caesalpinolida A and caesalpinolida B isolated from *C. bonduc* were reported to have cytotoxic activity against MCF-7 (Yadav et al., 2009). Also, brazilein isolated from *C. sappan* was reported active against HepG2 and Hep3B (liver), MDA-MB-231 and MCF-7 (breast), A549 (pulmonary), and CA9-22 (gingival) cells (Yen et al., 2010). 

ANOVA with Dunnett’s multiple test revealed that cytotoxicity of isolated compounds was not significantly different from that of methotrexate across the carcinoma cells. However, comparing the viability reducing ability of the isolated compounds with the negative control, the present study revealed that the isolated compounds elicited reduction in the number of viable cancer cells (Figure 5a-f), with varying potencies as reflected in their IC_50_ values (Tables 7 and 8). 

Investigation of *Caesalpinia benthamiana* leaves led to the identification of six compounds including four previously unreported (2, 3, 5, and 6) and two known ones (1 and 4). The compounds had significant cytotoxic effect against MCF-7, A549 and PC3. Overall, the diterpenoid, had the highest cytotoxic activity across the cells. The use of *C. benthamiana* in traditional medicine for the treatment of cancer, was affirmed by this study.
